# Monocyte-to-HDL Ratio (MHR) Predicts Vitamin D Deficiency in Healthy and Metabolic Women: A Cross-Sectional Study in 1048 Subjects

**DOI:** 10.3390/nu14020347

**Published:** 2022-01-14

**Authors:** Carlo De Matteis, Lucilla Crudele, Marica Cariello, Stefano Battaglia, Giuseppina Piazzolla, Patrizia Suppressa, Carlo Sabbà, Elena Piccinin, Antonio Moschetta

**Affiliations:** 1 Department of Interdisciplinary Medicine, University of Bari “Aldo Moro”, 70124 Bari, Italy; carlodematteis1991@gmail.com (C.D.M.); lucilla.crudele@uniba.it (L.C.); maricacariello@gmail.com (M.C.); battagliastefano87@gmail.com (S.B.); giuseppina.piazzolla@uniba.it (G.P.); patrizia.suppressa@uniba.it (P.S.); carlo.sabba@uniba.it (C.S.); 2 INBB, National Institute for Biostructures and Biosystems, 00136 Rome, Italy; 3 Department of Basic Medical Sciences, Neurosciences and Sense Organs, University of Bari “Aldo Moro”, 70124 Bari, Italy

**Keywords:** vitamin D, MHR index, metabolic syndrome, gender difference

## Abstract

Vitamin D deficiency is often linked with Metabolic Syndrome, both being more frequent with ageing and associated with an increase inflammatory state. Recently, monocytes-to-high density lipoprotein (HDL) ratio (MHR) has emerged as a powerful index to predict systemic inflammation. In this cross-sectional study, we investigated the association between circulating vitamin D level (25-OH vitamin D) and inflammatory status in a population of 1048 adult individuals. Our study reveals an inverse association between 25-OH vitamin D levels and MHR in the overall population. When the population is stratified by gender, waist circumference, and body mass index (BMI), we observed that while in men this relation is strongly significative only in condition of central obesity, in women a lifelong negative correlation exists between circulating 25-OH vitamin D and MHR and it is independent of the metabolic status. These observations underscore the relevance of circulating biomarkers such as MHR in the prediction of systemic inflammatory conditions sustained by vitamin D deficiency also in healthy and young women.

## 1. Introduction

Circulating low level of vitamin D is a common situation worldwide. Although the definition and relevance of this deficiency is still under debate [1,2], observational studies revealed that almost 40% of the European population present a deficit in vitamin D level (<20 ng/mL) [3], and more than 20% of people from India, Afghanistan, and Pakistan display a severe vitamin D depletion (<13 ng/mL) [4]. Obtainable with food or via skin synthesis after UVB exposure, vitamin D can be further metabolized in our organism by the liver, forming 25-hydroxyvitamin (25-OH vitamin D), the major circulating form, and then by the kidney into 1,25-dihydroxyvitamin, the active form of vitamin D. The latter can selectively bind to Vitamin D Receptor (VDR) and mediate the “non calcemic” effects of vitamin D. Indeed, besides the known role in calcium homeostasis, vitamin D is a recognized regulator of the endocrine and immune systems as well as adipose tissue functions [5].

Therefore, it is not surprising that the low serum vitamin D disposal may increase the risk of several diseases, including cancer, cardiovascular disease (CVD), and neurodegenerative disorders. These pathologies are frequently characterized by the existence of a latent chronic inflammation, which may contribute to the onset and severity of the diseases. Intriguingly, an inverse association between vitamin D levels and serum inflammatory markers, including interleukin-6, C-Reactive Protein (CRP), and Tumour Necrosis Factor α, has been recently discovered [6,7], thus suggesting that inflammation could be the common factor linking together illness and low vitamin D status [8].

Considering the importance of sub-inflammation in the development of Metabolic Syndrome (MetS), it has been hypothesized that vitamin D deficiency could play a contributory role in the development of this disorder. MetS is a condition characterized by different risk factors: central adiposity, dyslipidaemia, hypertension, hyperglycaemia, and low-grade inflammation. Similar to vitamin D, MetS also increases with ageing and has been reported to be widely implicated in the development of cancer and CVD.

Recently, the monocyte to high density lipoprotein ratio (MHR) has been addressed as a new indicator of subclinical inflammation, particularly elevated in CVD and useful in the prediction of MetS [9,10,11]. Monocytes represent the most important cells for the secretion of pro-inflammatory and pro-oxidants cytokines. Thereby, an increase in the quantity of monocytes is frequently associated with sub-inflammation [12]. By contrast, high density lipoprotein (HDL) generally exert an anti-inflammatory role, inhibiting monocytes’ activities and their differentiation into macrophages [13].

Despite several studies trying to disentangle the complex relationship between vitamin D deficiency and MetS, many of them failed to detect an association between the two conditions [14,15,16]. Moreover, although both vitamin D status and MetS display a different incidence in male and female population, just few studies have analysed the role played by gender difference in these two conditions [17,18].

Hereby, we analysed the vitamin D level in a population of 1048 healthy and metabolic subjects, taking in consideration gender difference and inflammatory status. Overall, our data depict an intriguing scenario in which gender difference is crucial in the determination of vitamin D level and its association with MHR.

## 2. Materials and Methods

### 2.1. Study Participants

Patients’ recruitments, clinical, and biochemical parameters were registered consecutively in the electronic health register of Metabolic Disease of Department of Interdisciplinary Medicine—Internal Medicine Division “Cesare Frugoni” of the “Aldo Moro” University of Bari, Policlinico (Bari, Italy) from January 2017 to July 2021. A total of 1819 patients were initially enrolled in this study, among which 465 were follow-up observations; therefore, they were excluded from the study population. To the remaining 1354 patients, 49 patients who referred having abused alcohol in the recent years were subsequently excluded. Then, we noticed that 51 out of the total 1354 patients lacked the value of waist circumference (WC) and/or blood count, thence we removed them from the study, reaching a number of 1254 patients. Patients with Inflammatory Bowel Disease and/or Coeliac disease (*n* = 12), acute heart diseases (cardiac failure, coronary arterial disease, acute arrhythmias), renal and hepatic failure, infections, secondary hypertension, chronic systemic inflammatory diseases, and neoplastic diseases with recent onset (less than 10 years) and/or under chemotherapy (*n* = 194) were excluded from the study. Finally, we performed statistical analysis on a population of 1048 patients (528 males, 520 females). The study was approved by the Ethics Committee (MSC/PBMC/15) of the Azienda Ospedaliero—Universitaria Policlinico di Bari (Bari, Italy) in accordance with the requirements of the Declaration of Helsinki. Written informed consent for the use of clinical data was obtained from all participants in the study.

### 2.2. Clinical Assessment

All participants underwent a detailed physical examination. Anthropometric assessment was performed using standardized procedures, as previously described [19]. Briefly, waist circumference (WC) was measured at the midpoint between the inferior part of the 12th costa and the anterior-superior iliac crests. BMI (body mass index) was computed as weight (kg) divided by the height (m) squared and BMI values (kg/m^2^) 25.0–29.9 and over 30.0 were considered as overweight and obesity conditions, respectively. Average systolic and diastolic blood pressure (BP) were derived for each patient from three measurements using manual sphygmomanometer. Hypertension was identified as systolic arterial blood pressure (SAP) ≥ 140 mmHg, diastolic arterial blood pressure (DAP) ≥ 90 mmHg and/or treatment with antihypertensive agents. Liver function tests like AST (SGOT) and ALT (SGPT) were analysed using cut-off limits of 37 U/L and 78 U/L, individually.

The cardiovascular risk was assessed using the official Framingham Heart Study calculator for cardiovascular disease in the upcoming 10 years adjusted for lipids. In accordance with the approved Ethics Committee, it was used to include patients with 18 or more years.

Prediabetes (preDM) and Diabetes Mellitus (DM) were identified according to international criteria suggested by the American Diabetes Association [20]. Specifically, PreDM was determined using the following criteria: HbA1c (percentage of glycosylated haemoglobin) ≥ 5.7% ≤ 6.4% and fasting plasma glucose (FPG) ≥ 100 ≤ 125 mg/dL. For DM, the criteria were: HbA1c (percentage of glycosylated haemoglobin) ≥ 6.5%, fasting plasma glucose (FPG) ≥ 126 mg/dL and/or treatment for diabetes. To characterize dyslipidaemia, HDL cut-off was <40 mg/dL in males and <50 mg/dL in females. Furthermore, a value of 150 mg/dL for both genders was considered pathological, whereas a total cholesterol level of ≥200 mg/dL was used for the diagnosis of hypercholesterolemia. The diagnosis of MetS was made according to 2006 IDF definition. The study population was also classified having examined the presence/absence of each criterion of MetS.

### 2.3. Biochemical Measurements

Morning blood samples were obtained after 12 h of fasting from the antecubital veins of patients. After blood clotting and centrifugation, serum was processed for analysis of biochemical markers of lipid and glucose metabolism. Renal, liver, inflammation thyroid function markers were as well studied following standardized biochemical procedures. All biochemical measurements were centralized and performed in the ISO 9001 certified laboratories of the University Hospital of Bari. Specifically, a complete blood count with determination of leukocyte’s subpopulation was performed. Measurements of total and HDL cholesterol, FPG, triglycerides were obtained through enzymatic colorimetric assay (Siemens, Erlangen, Germany). CPR via nephelometry (Siemens, Erlangen, Germany). C-peptide and 25-OH vitamin D were determined by CLIA on the LIAISON analyzer (DiaSorin, Inc., Stillwater, MN, USA). HbA1c was assessed in human whole blood using ion-exchange high-performance liquid chromatography (HPLC) on the Bio-Rad Variant II Hemoglobin A1c Program (BIO-RAD Laboratories Srl, Milan, Italy). Uric acid was determined by the URCA method on the Dimension Vista System (Siemens Healthcare Diagnostic Products GmbH, Marburg, Germany). LDL cholesterol level was obtained using the Friedewald formula and MHR was calculated manually.

### 2.4. Statistical Analysis

Descriptive statistical analyses of study sample were performed, and their results were expressed as mean ± standard error of the mean (SEM) and frequencies (%), based on the variable considered. Specifically, comparisons of socio-demographic and clinical variables between two groups were conducted with the Student T-test (for continuous variables) and the Pearson χ^2^ test (for categorical variables). Analysis between more than two groups were performed through one-way analysis of variance (ANOVA) followed by Bonferroni post-hoc test, where required. The correlation between continuous variables was also analysed and estimated using Pearson’s Correlation Coefficient (r). *p*-values lower than 0.05 were considered significant. All analyses were performed using the NCSS 12 Statistical Software, version 12.0.2018 (NCSS, LLC Company, Kaysville, UT, USA) and GraphPad Prism, version 9.1.0 (GraphPad Software; San Diego, CA, USA).

## 3. Results

### 3.1. Clinical Characterization of the Study Population

**Figure 1:**
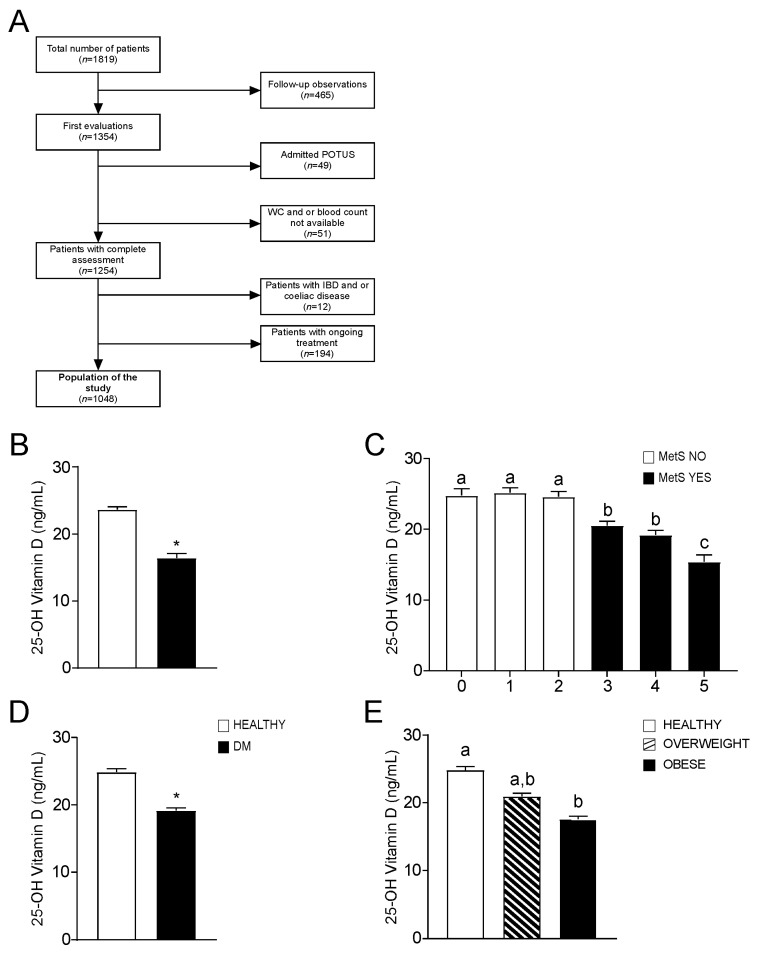
Flowchart of study population and 25-OH vitamin D levels in different subgroups. (**A**) Flowchart of the study population. (**B**) Comparison of 25-OH vitamin D levels in the entire study population. Subjects categorized based on Metabolic Syndrome (MetS) diagnosis; non-metabolic (MetS NO) subjects *n* = 466, MetS (MetS YES) patients *n* = 582. (**C**) Comparison of 25-OH vitamin D levels. Subjects were categorized based on positivity for MetS criteria. 0 criteria *n* = 139, 1 criterion *n* = 157, 2 criteria *n* = 170, 3 criteria *n* = 225, 4 criteria *n* = 216, 5 criteria *n* = 141. (**D**) Comparison of 25-OH vitamin D levels according to the type-2 Diabetes Mellitus (DM) definition (**E**) Comparison of 25-OH vitamin D values according to BMI definition. Subjects were divided in three different categories. Healthy *n* = 384, Overweight *n* = 341, Obese *n* = 323. All data are presented as mean ± SEM. Student T-test was performed for comparison of two groups, with * *p* < 0.05. One-way ANOVA test followed by Bonferroni’s post-hoc test was used for comparison of three or more groups. Data from groups sharing the same lowercase letter were not significantly different, whereas data from groups with different case letter were significantly different (*p* < 0.05).

Figure 1A represents a flow diagram that describes the process of selection of the population. We enrolled 1048 participants, whose baseline characteristics are presented in Table 1. Of these, 582 patients (340 males and 242 females) were diagnosed with Metabolic Syndrome (MetS), while 466 (188 males and 278 females) were non-metabolic subjects (MetS NO). Compared to the latter, MetS patients were older and exhibited increased weight, WC, BMI, systolic blood pressure, glycemia, HbA1c, triglycerides, and decreased HDL (*p* < 0.05). Surprisingly, no significant differences were found for inflammation markers (hs-PCR, ESR, WBC, neutrophils, monocytes, lymphocytes) in the two groups, whereas MetS patients showed significantly lower levels of 25-OH vitamin D compared to no-MetS subjects (*p* < 0.05). Considering non-invasive indexes, MetS patients presented increased cardiovascular risk (Framingham Score) and HOMA index ratio.

### 3.2. Association of Vitamin D Status with Metabolic Syndrome

To investigate the link between vitamin D levels and metabolic status, we compared 25-OH vitamin D levels in MetS and no MetS patients, showing that MetS subjects have significantly lower values (Figure 1B). Moreover, we observed that 25-OH vitamin D decreases when the number of positive diagnostic criteria of MetS increases (Figure 1C).

Given the role of MetS in diabetes pathogenesis [21], we analysed 25-OH vitamin D status also in diabetic patients and detected significantly lower levels of 25-OH vitamin D in diabetic subjects compared to non-diabetic ones (Figure 1D). Furthermore, since BMI is closely related to metabolic status, we examined the relation between BMI and 25-OH vitamin D, finding that 25-OH vitamin D levels decrease in overweight and obese patients (according to their BMI) compared to normal subjects (Figure 1E).

**Figure 2:**
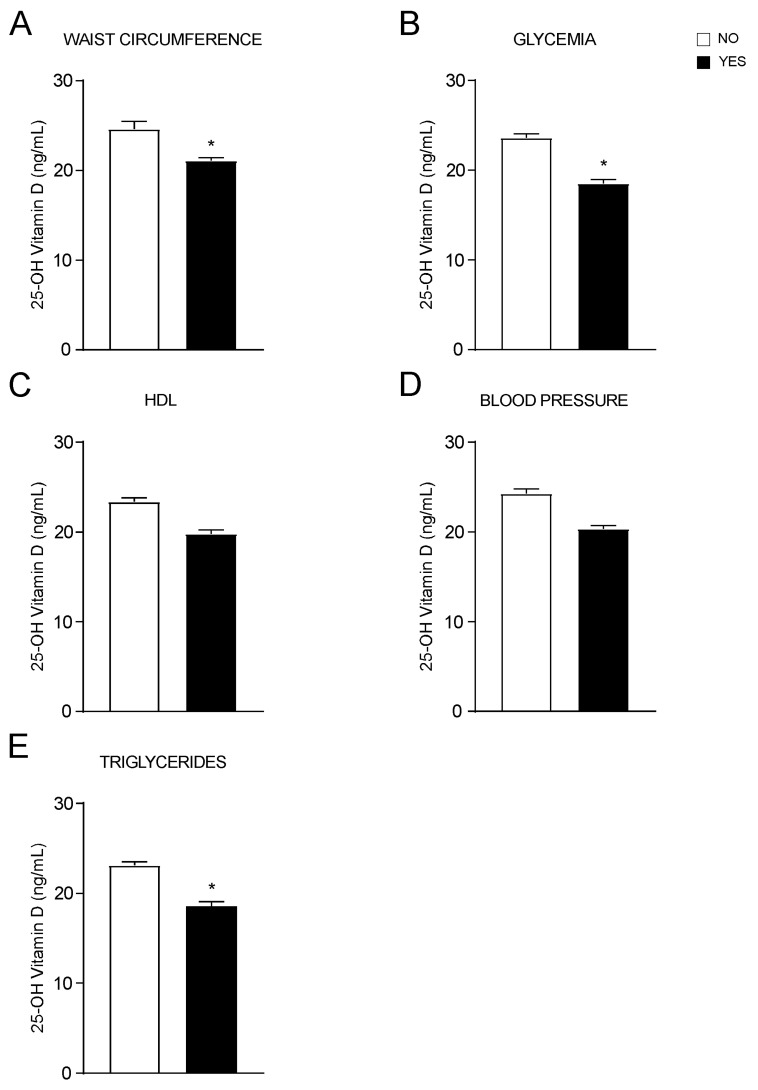
OH-25 vitamin D levels analysed by each MetS criterion. (**A**–**E**). T-Test comparison of OH-25 vitamin D values in subjects divided by positivity for each MetS criterion according to IDF definition. Data is presented as mean ± SEM. * *p* < 0.05.

Then, we assessed each diagnostic criterion of MetS individually, finding that 25-OH vitamin D significatively decreases in patients with higher WC (Figure 2A), in those with fasting hyperglycaemia or currently in treatment for diabetes (Figure 2B) as well as in patients with hypertriglyceridemia or under treatment for it (Figure 2E). No statistical differences in 25-OH vitamin D levels were observed in patients with low HDL (Figure 2C) and increased blood pressure (Figure 2D) compared to controls. These data confirm the relationship between vitamin D status and MetS, and its criteria. Particularly, to each criterion that is positively associated with MetS corresponds a decrease in vitamin D levels.

**Figure 3:**
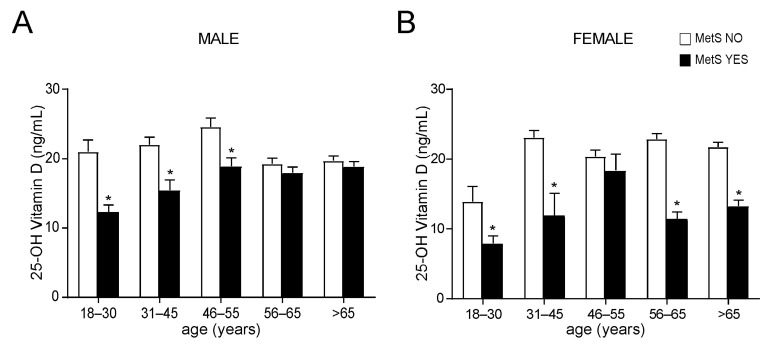
Comparison of OH-25 vitamin D levels in non-metabolic and metabolic subjects, divided by gender and age. (**A**) Comparison of OH-25 vitamin D levels in male subjects. Patients were classified in non-metabolic (MetS NO) and metabolic (MetS YES) subjects and divided by age ranges. Data is presented as mean ± SEM. Statistical significance was assessed by Student T-test (* *p* < 0.05). (**B**) Comparison of OH-25 vitamin D levels in female subjects according to the MetS definition (IDF). Patients were divided by age ranges. Data is presented as mean ± SEM. Statistical significance was assessed by Student *t*-test (* *p* < 0.05).

To better understand if age and/or gender could determine 25-OH vitamin D variations, we divided our study population by gender and then by age, identifying five different age ranges. The results showed that in all age ranges, MetS patients had lower levels of vitamin D with a significant reduction in younger males between the age of 18 and 55 years old (Figure 3A) and in every time of life in females (Figure 3B). Taken together, these data show that 25-OH vitamin D levels lifelong correlate with metabolic status, especially in females.

### 3.3. Vitamin D and MHR Correlation

Since inflammation is a hallmark of MetS [21] and vitamin D levels are strictly correlated both to inflammation and MetS [22], we evaluated if inflammation markers change in MetS patients according to their vitamin D status. To this end, the populations of MetS and no-MetS patients was divided in three groups based on their 25-OH vitamin D serum concentrations.

**Figure 4:**
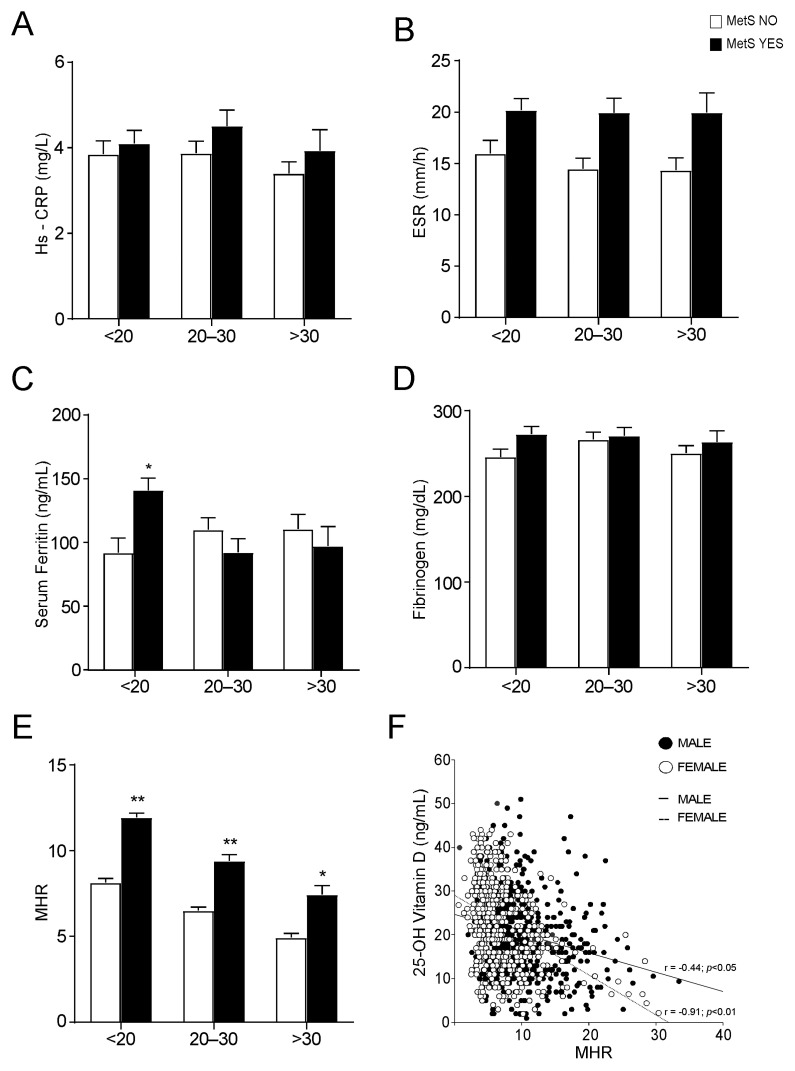
Evaluation of serum inflammation markers and Monocyte-to-HDL ratio in non-metabolic and metabolic subjects, divided by 25-OH vitamin D levels. (**A**–**D**) Comparison of serum inflammation markers in non-metabolic (MetS NO) and MetS (MetS YES) subjects, divided by 25-OH vitamin D levels. Data is presented as mean ± SEM. Statistical significance was assessed by Student T-test (* *p* < 0.05). (**E**) Comparison of Monocyte-to-HDL ratio (MHR) levels in non-metabolic (MetS NO) and MetS (MetS YES) subjects, divided by 25-OH vitamin D levels. Data is presented as mean ± SEM. Statistical significance was assessed by Student *t*-test (* *p* < 0.05, ** *p* < 0.01). (**F**) Correlation analysis of 25-OH vitamin D and MHR in the entire study population, divided by gender.

**Figure 5:**
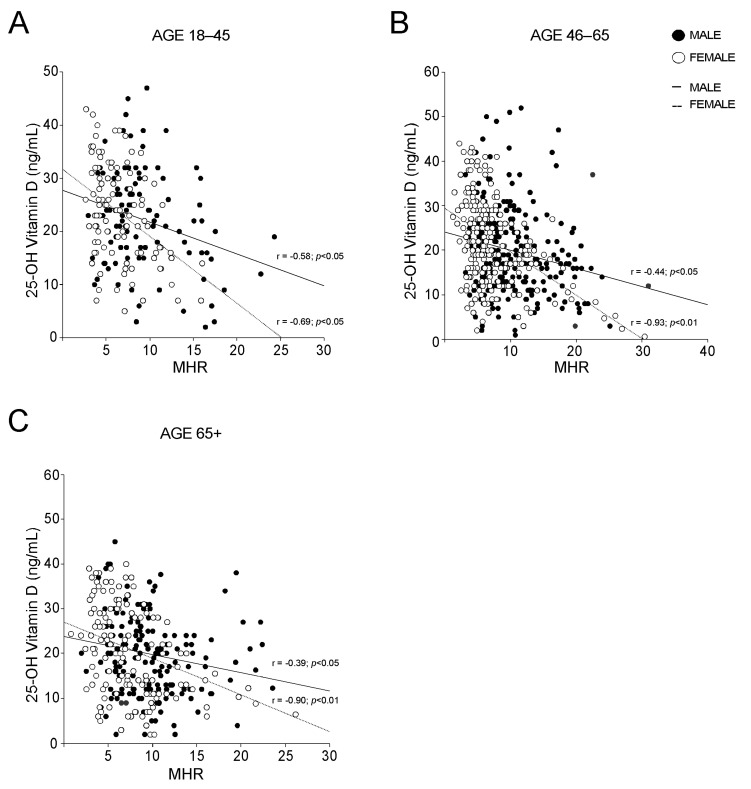
Gender-based correlation between 25-OH vitamin D and Monocyte-to-HDL ratio in different groups. (**A**–**C**) Correlation analysis of 25-OH vitamin D and MHR in male and female subjects; (**A**) analysis was conducted on subjects with age 18–45, (**B**) was conducted on subjects with age 46–65 and (**C**) was conducted on subjects with age 65+.

The analysis of the levels of different markers of inflammation revealed no significative differences among MetS and no-MetS subjects for hs-CRP, ESR, serum ferritin, and fibrinogen, apart from serum ferritin in vitamin D deficit (Figure 4A−D). By contrast, the statistical comparison of MHR in MetS and no-MetS individuals showed a significant upregulation of MHR values in MetS, for all vitamin D levels (Figure 4E). Interestingly, we detected a clear negative correlation trend: MHR values decrease correspondingly with vitamin D levels increase. To understand the significance of this observation, especially in relation to gender difference, we divided our cohort of patients by gender and studied the correlation between 25-OH vitamin D and MHR in both male and female (Figure 4F). As expected, we found a significative correlation in both genders, even if this correlation had higher statistical relevance in females (*p* < 0.01) than in males (*p* < 0.05). Interestingly, the correlation remained also after breaking out these two subpopulations by age (Figure 5). Similarly to what observed for MetS and vitamin D status, the correlation between vitamin D and MHR was statistically significant at any age considered. Anyway, by correcting this analysis by gender and age, we highlighted a stronger correlation in females (*p* < 0.01) older than 45 years old (Figure 5B,C) than in matched age males (*p* < 0.05).

**Figure 6:**
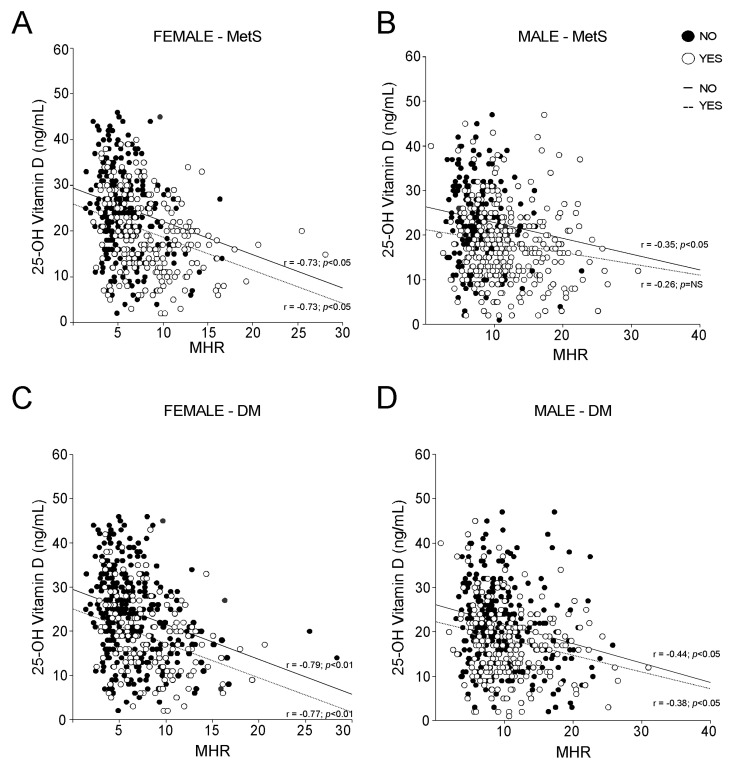
Correlation between 25-OH vitamin D and Monocyte-to-HDL ratio according to MetS and Diabetes Mellitus (DM) definitions. (**A**,**B**) Correlation analysis of 25-OH vitamin D and MHR in male and female subjects, divided by non-metabolic status (NO) and MetS diagnosis (YES). (**C**,**D**) Correlation analysis of 25-OH vitamin D and MHR in male and female subjects, divided by absence (NO) and presence (YES) of Diabetes Mellitus diagnosis.

To evaluate the influence of metabolic disorders on vitamin D and MHR relation, we analysed MHR and vitamin D in MetS compared to non-MetS females (Figure 6A) and males (Figure 6B). Our statistical analyses showed that the correlation between MHR and vitamin D is independent from MetS in females, while in males this correlation is lost in MetS patients. On the contrary, we found that the presence of diabetes did not influence the relationship between MHR and vitamin D in both genders, although a better significance has been observed in females (Figure 6C) with respect to males (Figure 6D).

**Figure 7:**
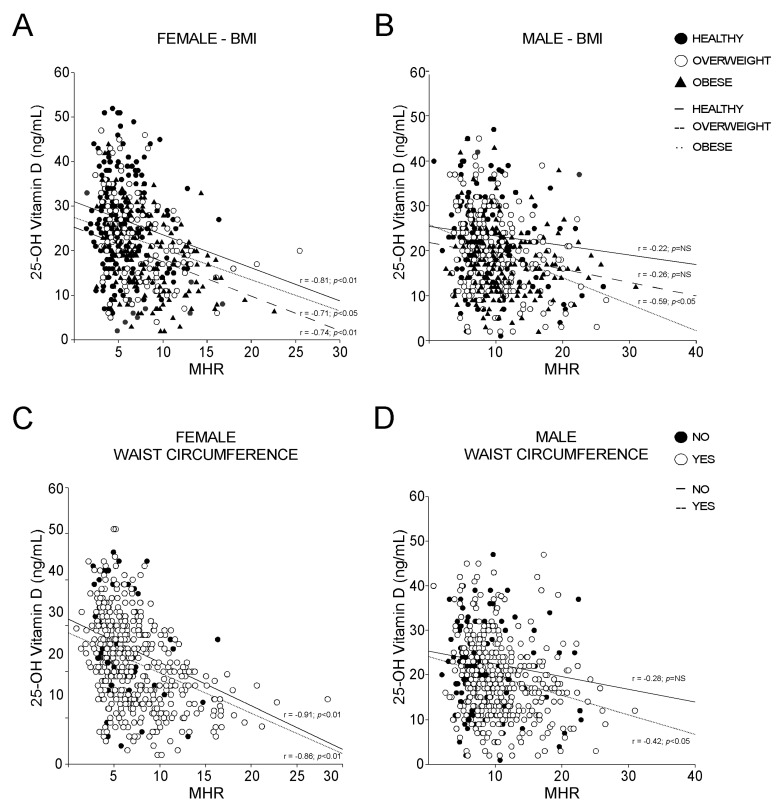
Correlation between 25-OH vitamin D and Monocyte-to-HDL ratio according to BMI definition and waist circumference criterion for MetS. (**A**,**B**) Correlation analysis of 25-OH vitamin D and MHR in female and male subjects, divided according to BMI levels in healthy, overweight, and obese. (**C**,**D**) Correlation analysis of OH-25 vitamin D and MHR in female and male subjects, classified by absence (NO) and presence (YES) of positive waist circumference criterion for MetS.

To deepen our analysis, we stratified the populations by BMI and WC, and the strength of the MHR-vitamin D correlation in females was dramatically clear. Intriguingly, the vitamin D-MHR correlation in female is independent from BMI (Figure 7A) and WC (Figure 7C), always showing very high statistical significance (*p* < 0.01). By contrast, in male, this relationship was detected only in patients defined as obese by BMI (Figure 7B) or with increased WC (Figure 7D), but with lower statistical strength than in females (*p* < 0.05). Taken together, these findings confirm that a relation between vitamin D and MHR may exist.

## 4. Discussion

This cross-sectional population-based study provides evidence that 25-OH vitamin D inversely correlates with MHR index. Particularly, this correlation has an extreme significance lifelong in females, regardless their metabolic state. Conversely, a significant inverse relationship between 25-OH vitamin D and MHR is observed in males that are obese or that display an increased visceral adiposity.

Vitamin D metabolism is closely related to body fat content [23], and its levels are negatively associated with increased waist circumference and BMI [24]. Different hypotheses raised until now have tried to explain the nexus between adiposity and vitamin D. Firstly, adipose tissue is the main storage depot for vitamin D, given the fat soluble nature of this molecule, and functions as a buffering system that slowly releases vitamin D in the circulation during fasting, thus avoiding unregulated production of functional 25-OH Vitamin D after dietary intake [25,26]. Then, the limited mobility that characterized many overweight and obese subjects may result in a decrease exposure to sunlight, hence negatively impairing the vitamin D synthesis [27].

Besides being sequestered by the adipose tissue, vitamin D can also play different roles in fat metabolism. Vitamin D seems to modulate some of the genes involved in the regulation of serum cholesterol via VDR [28,29]. Indeed, vitamin D levels positively correlates with HDL, especially in male population [30,31]. However, it is still not clear if also the supplementation of vitamin D may increase the level of HDL cholesterol [32,33,34].

Furthermore, vitamin D is involved in the adipogenesis, regulating multiple levels of the differentiation process of pre-adipocytes in mature adipocytes [35,36,37,38]. Intriguingly, low levels of vitamin D have been associated with increase in low grade inflammation, which can act as a stimulatory factor in promoting adipocytes proliferation and the remodelling of adipose tissue, especially in obesity [39,40]. Central obesity induces macrophages infiltration and activation in adipose tissue, causing a low grade inflammation. In this scenario, both adipocytes and adipose tissue-infiltrating macrophages are prominent source of pro-inflammatory cytokines, further exacerbating the damage [41]. Active metabolites of vitamin D act directly on macrophages, diminishing the release of proinflammatory cytokines and chemokines, therefore limiting sub-inflammatory state [42].

Our data display an inverse correlation between 25-OH-vitamin D and MHR. The latter has emerged as a powerful index of systemic inflammation that, integrating both proinflammatory (monocytes) and anti-inflammatory (HDL) factors, is useful in the prediction of different pathological conditions, especially cardiovascular ones [10,43,44]. Differently from Mousa et al., in our population, 25-OH-vitamin D levels are negatively associated not only in young individuals, but also in older ones [45]. Specifically, in our population, a stronger correlation between 25-OH-vitamin D and MHR is observed with the progression of age. Of note, vitamin D may interfere with both the parameters of MHR calculation. Indeed, besides playing a role in HDL formation, active vitamin D metabolites lower the release of proinflammatory mediators, thus impairing the recruitment of monocytes/macrophages and reducing overall inflammation [46,47].

Recently, an association between MHR and MetS was depicted [11,48,49,50], highlighting a gender influence in the determination of the index. In our study, the low levels of vitamin D correlate with MetS and its criteria (waist circumference, hyperglycaemia, and hypertriglyceridemia), predominantly in females. Given that vitamin D deficiency is closely related to body fat content and inflammation and that increased waist circumference has been associated with a sub-inflammatory state, it is possible to wonder if a supplementation of vitamin D to those subjects that display vitamin D deficiency together with increase MHR could exert a beneficial effect, protecting them from the onset of severe health consequences.

It is important to note that a gender difference exists in fat distribution between women and men. Generally, women display higher body fat than men, which is usually more present in the gluteal-femoral region, differently from men that store more fat in the visceral depot. Central obesity is mainly characterized by a low-grade inflammatory status that may promote the onset of diseases. This may explain why in our male population the inverse correlation between 25-OH vitamin D and MHR is observed only in those that are obese or with an increased waist circumference, while in females it is independent from the metabolic status. Consistently, previous studies indicate that vitamin D deficiency in obese individuals is given by limited bioavailability due to increased deposition, especially in visceral fat depots [51,52]. Therefore, these findings highlight the importance of considering the gender difference when determining the level of vitamin D and its association with MHR. In conclusion, by stratifying our population by gender, waist circumference, and body mass index (BMI), we observed that while in males this relation is strongly significative only in condition of central obesity, in females a lifelong negative correlation exists between circulating 25-OH vitamin D and MHR and it is independent of the metabolic status. Since other indexes of inflammation, such as peripheral blood neutrophil-to-lymphocyte ratio and platelet-to-lymphocyte ratio, have been recently proposed as a predictor of osteoporosis, a bone disease typical of menopausal women [53,54], it would be intriguing to understand if inflammation indexes as MHR could be useful to predict 25-OH vitamin D deficiency and the related clinical conditions. These observations underscore the relevance of circulating biomarkers such as MHR in the prediction of systemic inflammatory conditions sustained by vitamin D deficiency also in healthy and young women.

## Figures and Tables

**Table 1:** Clinical characterization of the study population ^1^.

Clinical Variable	Healthy	MetS	*p*-Value
*n* (M:F)	466 (188:278)	582 (340:242)	-
Age (years)	50.01 ± 0.73	60.71 ± 0.48	<0.05
Weight (Kg)	70.45 ± 0.75	83.10 ± 0.74	<0.05
Waist circumference (cm)	91.33 ± 0.64	105.96 ± 0.55	<0.05
BMI (Kg/m^2^)	25.25 ± 0.24	30.07 ± 0.23	<0.05
Sistolic blood pressure (mmHg)	120.68 ± 0.68	134.35 ± 0.66	<0.05
Diastolic blood pressure (mmHg)	76.02 ± 0.46	80.45 ± 0.42	NS
Platelet count (10^6^/μL)	240.52 ± 2.86	232.42 ± 2.75	NS
Hemoglobin (g/dL)	13.69 ± 0.07	13.95 ± 0.07	NS
WBC (10^3^/µL)	5.92 ± 0.08	6.90 ± 0.08	NS
Monocytes (%)	6.21 ± 0.07	6.38 ± 0.07	NS
Lymphocytes (%)	32.83 ± 0.35	31.03 ± 0.32	NS
Neutrophils (%)	57.64 ± 0.38	59.28 ± 0.34	NS
Basophils (%)	0.56 ± 0.01	0.56 ± 0.02	NS
Eosinophils (%)	2.65 ± 0.08	2.76 ± 0.08	NS
Glucose (mg/dL)	86.95 ± 0.55	117.97 ± 1.66	<0.05
HbA1c (mmol/mol)	35.10 ± 0.37	46.61 ± 0.66	<0.05
Total cholesterol (mg/dL)	188.93 ± 1.69	179.16 ± 1.96	NS
HDL-c (mg/dL)	59.77 ± 0.64	47.09 ± 0.58	<0.05
LDL-c (mg/dL)	111.13 ± 1.49	100.10 ± 1.49	NS
TG (mg/dL)	90.30 ± 1.74	158.88 ± 3.66	<0.05
AST (U/I)	22.94 ± 0.44	25.55 ± 0.53	NS
ALT (U/I)	28.89 ± 0.64	34.95 ± 0.97	NS
ALP (U/I)	69.14 ± 1.09	73.51 ± 1.17	NS
GGT (U/I)	28.81 ± 1.19	39.59 ± 1.65	<0.05
Creatinine (mg/dL)	0.78 ± 0.01	0.86 ± 0.01	NS
Uric acid (mg/dL)	4.09 ± 0.06	4.89 ± 0.07	NS
25-OH vitamin D(ng/mL)	24.54 ± 0.47	19.00 ± 0.37	<0.05
Total protein (g/dL)	7.31 ± 0.04	7.31 ± 0.02	NS
Albumin (g/dL)	4.44 ± 0.02	4.41 ± 0.02	NS
ESR (mm/h)	14.81 ± 0.67	20.06 ± 0.79	NS
Hs-CRP (mg/L)	3.71 ± 0.16	4.2 ± 0.21	NS
TSH (mUI/L)	1.82 ± 0.08	2.08 ± 0.09	NS
FT3 (pg/mL)	2.85 ± 0.03	2.77 ± 0.03	NS
FT4 (ng/dL)	1.05 ± 0.02	1.05 ± 0.01	NS
Cardiovascular risk (Framingham)	9.74 ± 0.54	29.24 ± 0.79	<0.05
HOMA index ratio	1.91 ± 0.10	3.88 ± 0.23	<0.05

^1^ Data are presented as mean ± SEM (standard error of the mean). BMI Body Mass Index, WBC White Blood Cell Count, HbA1c glycosylated hemoglobin, TC total cholesterol, HDL-C high-density lipoprotein cholesterol, LDL-C low-density lipoprotein cholesterol, TG triglyceride, AST aspartate transaminase, ALT alanine transaminase, ALP alkaline phosphatase, GGT gamma-glutamyltransferase, Hs-CRP high-sensitivity C reactive protein, TSH thyrotropin, FT3 tri-iodothyronine, FT4 thyroxine.

## Data Availability

The data presented in this study are available on request from the corresponding author. The data are not publicly available due to ethical issues.
